# 小细胞肺癌的免疫特征

**DOI:** 10.3779/j.issn.1009-3419.2020.101.33

**Published:** 2020-10-20

**Authors:** 燕 朱, 世凯 吴

**Affiliations:** 100034 北京，北京大学第一医院肿瘤化疗科 Department of Oncology, Peking University First Hospital, Beijing 100034, China

**Keywords:** 小细胞肺癌, 免疫检查点抑制剂, 肿瘤免疫微环境, Small cell lung cancer, Immune checkpoint inhibitor, Tumor immune microenvironment

## Abstract

小细胞肺癌（small cell lung cancer, SCLC）是预后极差的一类肿瘤，30年来药物治疗无显著进展，免疫检查点抑制剂（immune checkpoint inhibitor, ICI）成为近年唯一突破：程序性死亡-1（programmed death-1, PD-1）抑制剂单药或联合细胞毒T淋巴细胞抗原-4（cytotoxic T-lymphocyte antigen-4, CTLA-4）抑制剂后线治疗SCLC的有效率为10%-33%，有效时间较持久；程序性死亡配体-1（programmed death ligand-1, PD-L1）抑制剂联合化疗对比传统化疗一线治疗广泛期SCLC（extensive stage-SCLC, ES-SCLC）的总生存期延长。尽管取得一定疗效，相对于非小细胞肺癌（non-small cell lung cancer, NSCLC）等对免疫治疗敏感的肿瘤类型，SCLC的疗效仍不令人满意，这可能与其免疫抑制特征有关。本综述对SCLC免疫特征的研究现状进行总结，包括淋巴细胞和免疫抑制细胞在肿瘤内浸润情况、PD-L1和主要组织相容复合物（major histocompatibility complex, MHC）在肿瘤的表达以及外周血免疫细胞的改变，并对这些免疫特征的预后及其对ICI疗效的预测价值进行分析。

## Abstract (en)

Small cell lung cancer (SCLC) is a type of malignancy with poor prognosis, and no advance in medication has been made for about 30 years except immune checkpoint inhibitor (ICI), which demonstrated efficacy in recent years. The response rate of programmed death-1 (PD-1) inhibitor alone or its combination with cytotoxic T-lymphocyte antigen-4 (CTLA-4) inhibitor as subsequent therapy was 10%-33% and the response duration was persistent. The combination of programmed death ligand-1 (PD-L1) inhibitor with chemotherapy resulted in longer survival versus chemotherapy alone. Nevertheless, comparing with immunotherapy-sensitive tumors such as non-small cell lung cancer (NSCLC), efficacy in SCLC is still unsatisfied and this is maybe associated with its immune inhibitory characteristics. This review describes the current research about immune characteristics of SCLC, including tumor infiltrating of lymphocytes (TIL) and immune inhibitory cells, PD-L1 and major histocompatibility complex (MHC) expression in tumor as well as changes of peripheral immune cells. We also review the prognostic and predictive values of these immune characteristics.

## 1 小细胞肺癌（small cell lung cancer, SCLC）的免疫治疗进展和存在的问题

肺癌是发病率和死亡率最高的实体肿瘤类型^[1]^，其中，SCLC约占肺癌总发病率的10%^[1]^，其特点为恶性度高、生长速度快、早期易发生转移，预后极差。SCLC的药物治疗30年来没有突破^[1]^，免疫治疗方面也进行了大量的尝试包括干扰素、肿瘤疫苗等均未显示明确获益^[2, 3]^。

免疫检查点抑制剂（immune checkpoint inhibitor, ICI）是近年来肿瘤治疗领域的新型免疫药物，在多种类型恶性肿瘤证实有效。免疫检查点属于免疫抑制型受体，包括程序性死亡-1（programmed death-1, PD-1）、细胞毒T淋巴细胞抗原-4（cytotoxic T-lymphocyte antigen-4, CTLA-4）、淋巴细胞活化基因3（lymphocyte activation gene-3, LAG-3）和T细胞免疫球蛋白黏蛋白分子-3（T cell immunoglobulin and mucin-domain containing-3, TIM-3）等。PD-1表达于T、B淋巴细胞、自然杀伤细胞、树突状细胞和单核细胞，生理状态下维持正常组织的免疫耐受^[4]^。PD-1与其配体程序性死亡配体-1（programmed death ligand-1, PD-L1）和PD-L2结合后抑制T细胞受体（T-cell receptor, TCR）信号途径，从而抑制T细胞功能^[4]^。肿瘤细胞和/或肿瘤组织中的淋巴细胞、单核-巨噬细胞、树突状细胞等免疫细胞通过表达PD-L1抑制抗肿瘤免疫反应，而PD-1或PD-L1抑制剂能够重新激活受抑制的T细胞^[4]^，现已被多个指南列为晚期实体肿瘤包括肺癌的标准治疗药物。

SCLC具有的某些特征提示其可能对ICI敏感。SCLC属于具有高肿瘤突变负荷的瘤种，Chalmers等^[5]^对913例标本的检测显示平均突变数为9.9个突变/Mb。合并神经副瘤综合征的SCLC患者肿瘤进展慢于不合并神经副瘤综合征患者^[6]^，肿瘤异位表达神经系统特异性抗原，诱导机体产生抗瘤免疫反应可能是潜在机制^[7]^。另外，有研究显示SCLC患者外周血效应T细胞或淋巴细胞数目与长期生存相关^[8]^，外周血CD8^+^ T细胞增殖与患者治疗后更长的无进展生存期（progression-free survival, PFS）相关^[9]^。这些现象提示一部分SCLC患者体内可能存在天然抗肿瘤免疫应答，而ICI可能会增强这一效应。

令人鼓舞的是，PD-(L)1抑制剂确实也成为SCLC近年来药物治疗的唯一突破：Ⅰ期/Ⅱ期多中心CM032研究显示含铂化疗方案进展的SCLC患者使用PD-1抑制剂纳武利尤单抗（联合或不联合CTLA-4抑制剂依匹木单抗）的总有效率（overall response rate, ORR）为10%-33%^[10]^；对Ib期KN028和Ⅱ期KN158研究的合并分析显示既往治疗进展的SCLC患者使用PD-1抑制剂帕博利珠单抗的ORR为19.3%^[11]^。这两项研究^[10, 11]^均显示出ICI疗效持久的特点。据此，这两种治疗方案已被美国国家综合癌症网络（National Comprehensive Cancer Network, NCCN）指南列为SCLC患者的后线标准治疗药物。另外，两项Ⅲ期研究显示PD-L1抑制剂联合化疗一线治疗广泛期SCLC（extensive-stage small cell lung cancer, ES-SCLC）的疗效优于单纯化疗：在IMpower133研究中，阿替利珠单抗联合化疗的总生存期（overall survival, OS）为12.3个月，死亡风险较单纯化疗减少30%^[12]^；在CASPIAN研究中，度伐利尤单抗联合化疗与单纯化疗相比也显著延长OS，死亡风险下降27%^[13]^。据此，这两种治疗方案被NCCN指南列为ES-SCLC的一线标准治疗方案。而CTLA-4抑制剂联合化疗一线治疗ES-SCLC的OS、PFS和反应持续时间（duration of response, DOR）在Ⅲ期研究中不优于化疗^[14]^。其他免疫检查点包括LAG-3和TIM-3等的抑制剂正在进行临床研究^[15]^。

虽然PD-(L)1抑制剂在SCLC有效患者的疗效持久，但总体疗效仍明显差于非小细胞肺癌（non-small cell lung cancer, NSCLC）这类对免疫治疗敏感的肿瘤：纳武利尤单抗对比化疗治疗复发或进展SCLC的Ⅲ期CheckMate331研究未能显示OS延长^[16]^；阿替利珠单抗对比化疗二线治疗复发SCLC的Ⅱ期IFCT-1603研究未显示ORR、PFS和OS的改善^[17]^；度伐利尤单抗后线治疗ES-SCLC的ORR只有9.5%^[18]^；PD-L1抑制剂联合化疗比较化疗一线治疗ES-SCLC时，平均生存时间的延长有限^[12, 13]^。这可能与SCLC具有的一些免疫抑制特征相关。根据现有的研究结果，我们对相关领域研究进行如下总结。

## 2 SCLC具有的免疫特征

SCLC存在以下免疫抑制特征：①肿瘤组织中的浸润淋巴细胞（tumor infiltrating lymphocyte, TIL）少；②肿瘤内存在抑制型免疫细胞如肿瘤相关巨噬细胞（tumor associated macrophage, TAM）、髓系衍生抑制细胞（myeloid-derived suppressor cell, MDSC）；③肿瘤组织PD-L1表达阳性率低，且现有的临床研究尚不明确其对ICI的疗效预测价值；④肿瘤细胞主要组织相容性复合体（major histocompatibility complex, MHC）表达水平低；⑤患者外周血免疫细胞发生利于肿瘤生长的改变。

### 2.1 SCLC肿瘤浸润淋巴细胞水平低

Carvajal-Hausdorf等^[19]^对90例SCLC标本的检测显示TIL种类主要为CD3^+^/CD8^+^ T淋巴细胞和CD20^+^ B淋巴细胞，不同个体CD3、CD8和CD20的表达水平跨度大，平均表达水平低，16%的患者CD8和CD20均不表达。与NSCLC比较，表达水平最低的是CD8：比肺腺癌低5.4倍，比肺鳞癌低6倍，CD8/CD3比值也显著低于NSCLC，说明SCLC有较少的细胞毒性T细胞（cytotoxic T lymphocyte, CTL）浸润。与其他多种类型肿瘤不同的是，SCLC肿瘤CD8^+^ T细胞浸润程度与预后无关^[19, 20]^，这可能是因为CD8^+^细胞数量过低导致^[19]^。这些现象提示大部分SCLC属于免疫冷肿瘤^[19]^。

### 2.2 SCLC肿瘤中免疫抑制细胞浸润情况

#### 2.2.1 肿瘤相关巨噬细胞

巨噬细胞是肿瘤浸润免疫细胞（tumor infiltrating immune cell, TIIC）的重要组成成员，称为TAM。TAM来源于MDSC或单核细胞，其具有促进肿瘤生长、转移、血管生成和免疫抑制功能^[21]^。有研究^[21]^显示MDSC浸润肿瘤后，在低氧环境中被快速诱导分化为TAM，后者通过表达PD-L1等机制具有更广泛的T细胞抑制作用。TAM不仅表达PD-L1，还表达PD-1，PD-1抑制了TAM对肿瘤细胞的吞噬功能，与肿瘤进展相关^[22]^。因此，TAM是影响肿瘤固有免疫和适应性免疫的重要环节。

多项研究^[23-26]^显示TAM同样也是SCLC TIIC的重要组成。SCLC患者的外周血肿瘤细胞能够在体外诱导单个核细胞向TAM分化^[27]^。Gadgeel等^[24]^的研究显示8例/20例使用帕博利珠单抗维持治疗的SCLC患者瘤巢周围间质的PD-L1表达阳性，以巨噬细胞为主，PD-L1阳性患者显示更长的PFS和OS，提示SCLC肿瘤中的TAM可能抑制抗肿瘤免疫反应，但由于研究纳入人数较少，尚需在更大规模研究中进行探讨。有趣的是，早期的一项研究^[25]^显示与不合并神经副瘤综合征Lambert-Eaton肌无力综合征（Lambert-Eaton Myasthenic Syndrome, LEMS）的SCLC患者相比，合并LEMS患者（肿瘤进展慢）的肿瘤中有更多激活的巨噬细胞浸润，提示存在强烈的抗肿瘤免疫反应。巨噬细胞在不同肿瘤免疫微环境中的表型和作用可能有很大差别，因此需要进一步研究SCLC中不同TAM亚型与患者预后及免疫治疗疗效之间的关系。

#### 2.2.2 髓系衍生抑制细胞

MDSC由一群具有异质性的不成熟髓系细胞组成。肿瘤产生多种因子促进MDSC生成并浸润肿瘤，肿瘤还通过多种因子如血管内皮生长因子、粒细胞集落刺激因子、粒细胞-巨噬细胞集落刺激因子和白细胞介素-6等维持MDSC增殖和活性^[28]^。MDSC能通过多种机制如产生高水平的氧化氮、上调精氨酸酶（arginase-1, ARG-1）、表达PD-L1等抑制T细胞增殖，导致T细胞失活和凋亡；抑制NK细胞的活性；诱导CD4^+^ T细胞向调节性T细胞（regulatory T cell, Treg）分化和增殖；诱导巨噬细胞向促进肿瘤生长的表型分化^[21, 28, 29]^。还有证据^[29]^显示MDSC在各种类型肿瘤中起着重要的免疫抑制作用，影响免疫治疗疗效。

MDSC可能也是SCLC免疫抑制机制之一。Tian等^[30]^发现SCLC患者外周血MDSC的数量和比例显著高于正常人，高MDSC比例还与分期晚和更差的预后相关。程颖等^[31]^的研究也显示相似的结果。Iclozan等^[32]^探讨了SCLC患者使用具有MDSC抑制作用的全反式维甲酸对p53疫苗诱导的免疫反应的影响，结果显示维甲酸联合疫苗组和单独使用疫苗组机体的p53特异性反应率有显著差异，维甲酸提高了颗粒酶B阳性CD8^+^ T细胞在肿瘤中的浸润，提示MDSC参与抑制SCLC患者的抗瘤免疫反应，而MDSC抑制剂有可能提高免疫治疗的疗效。

### 2.3 SCLC肿瘤中免疫细胞分布位置及其意义

Hamilton等^[33]^认为SCLC标本中的PD-1阳性TIL多分布于肿瘤和间质交界处，并没有浸润肿瘤内部，这可能是SCLC对免疫治疗疗效不佳的原因之一。对其他瘤种TIIC分布的研究显示：PD-(L)1抑制剂治疗前，TIIC在肿瘤边缘分布不一定代表对免疫治疗疗效差，甚至可能代表效果更佳，如Tumeh等^[34]^对免疫治疗前和治疗中的黑色素瘤标本的检测显示：有效患者在用药前的肿瘤侵犯边缘和肿瘤内部含有更多的PD-1阳性CD8^+^ T细胞和PD-L1阳性肿瘤细胞或免疫细胞，PD-1阳性和PD-L1阳性细胞的位置密切相关，作者推测这是肿瘤针对内源性抗瘤免疫反应产生适应性抵抗的表现^[34]^。有效患者治疗中肿瘤内部和边缘的CD8^+^ T细胞浸润增加。其中，治疗前肿瘤边缘CD8^+^ T细胞浸润密度对于疗效判断最准确。Kuang等^[35]^的研究显示肝细胞癌瘤巢周边而不是瘤巢内部的巨噬细胞表达的PD-L1介导免疫抵抗。Korehisa等^[36]^比较了微卫星高度不稳定性[PD-(L)1抑制剂疗效好]和微卫星稳定性[PD-(L)1抑制剂疗效差]结直肠癌标本，发现前者肿瘤边缘的肿瘤细胞和巨噬细胞表达更高水平的PD-L1，并且肿瘤内有更多的CD8^+^ T细胞和巨噬细胞浸润。

各种TIIC亚群在SCLC中的分布模式也显示出类似特征。Schultheis等^[26]^发现患者肿瘤细胞均不表达PD-L1且TIL少有表达PD-1，但在肿瘤与间质交界处PD-L1和PD-1阳性率分别为18.5%和48%，阳性表达细胞分别为TAM和CD3^+^ T细胞，二者的分布位置密切。Yu等^[23]^的研究显示PD-L1在肿瘤-间质交界处的免疫细胞的表达常常与其他免疫指标同时出现，包括免疫细胞浸润程度、PD-L1在癌细胞表达。另一项小样本研究^[24]^提示PD-L1在瘤巢周边巨噬细胞表达时，患者使用PD-1抑制剂具有更高的有效率和更长的生存时间。

### 2.4 SCLC PD-L1表达特点及其意义

PD-L1在肿瘤细胞或肿瘤组织中的免疫细胞表达介导肿瘤发生免疫逃逸^[4]^，PD-L1在NSCLC、肾癌、黑色素瘤、膀胱癌等多种类型实体肿瘤的表达与PD-(L)1抑制剂更好的疗效相关^[37]^。

#### 2.4.1 PD-L1在SCLC肿瘤细胞的表达

不同研究检测PD-L1在SCLC肿瘤细胞表达阳性率跨度大：0%-82.8%，多数（13/17）研究显示PD-L1阳性率低：0%-25%，明显低于NSCLC（表 1）。

**1:** 小细胞肺癌PD-L1表达的相关研究 Research of PD-L1 expression in small cell lung cancer

Cases No.	PD-L1 expression		CPS	Relationship with other immune biomarkers	Prognostic or predictive value	Ref.
	TC (%)	IC (%)				
94^[a]^	0	18.5	-	PD-L1^+^ TAMs had close geographic association with PD-1^+^ T cells	ND	^[26]^
194	16.5^[b]^	56.3 (SP142);44.8 (28-8)^[c]^	-	PD-L1 expression on TC or IC was associated with the degree of IC infiltration; PD-L1 expression on TC was associated with its expression on macrophage	ES-SCLC patients with positive PD-L1 expression on TC trended toward longer survival; PD-L1 expression on TC was not associated with disease stages	^[23]^
30	10.0	40.0	-	ND	PD-L1 expression on IC was associated with longer PFS and OS after PD-1 inhibitor continuation therapy	^[24]^
277	5.1	22.3	-	ND	PD-L1 expression on TC or IC was not associated with the efficacy of PD-L1 inhibitor combined with chemotherapy	^[41]^
104	25.0	40.4	-	PD-L1 expression on TC was positively associated with CD8^+^ T cells infiltrating; PD-L1 expression on IC was positively associated with FOXP3^+^ T cells infiltrating	Positive PD-L1 expression on TC or IC was associated with early-stage disease; and was associated with longer OS in univariate analysis but not in multivariate analysis	^[20]^
186	78.0	54.3	-	PD-L1 expression on TC was positively associated with its expression on TIL	PD-L1 expression on TC was associated with poor survial	^[40]^
32^[d]^	75.0	25.0 (TIL) 28.0 (TAM)	-	PD-L1 expression was not associated with TIL density	PD-L1 expression on TIL was associated with longer survival	^[43]^
90	7.3	ND	-	PD-L1 expression was not associated with TIL markers	PD-L1 expression was not associated with 5-year survival	^[19]^
74	18.9	ND	-	ND	PD-L1 expression was associated with longer survival	^[39]^
102	71.6	ND	-	ND	PD-L1 expression was associated with early-stage disease and longer survival in ES-SCLC	^[38]^
148	17.0 (cutoff≥1); 5.0 (cutoff≥5)	ND	-	ND	PD-L1 expression was not associated with response rate of PD-1 inhibitor	^[10]^
210	1.9	ND	-	PD-L1 expression was positively associated with IC infiltrating in tumor	ND	^[50]^
39	2.5	ND	-	ND	ND	^[51]^
100	82.8	ND	-	ND	ND	^[52]^
20	5.0	ND	-	ND	ND	^[53]^
145	-	-	31.7	ND	In patients with positive PD-L1 (CPS≥1), there was no correlation between higher PD-L1 expression and response rate	^[54]^
107	-	-	39.0	ND	Positive PD-L1 was associated with response rate, 1-year OS and PFS after PD-1 inhibitor therapy	^[44]^
[a] including 61 cases of small cell lung cancer and 43 cases of exrapulmonary small cell cancer; [b] PD-L1 expression in overall patients; [c] PD-L1 expression in patients with limited-stage small cell lung cancer; [d] patients with brain metastasis. PD-L1: programmed death ligand-1; PD-1: programmed death-1; TC: tumor cell; IC: immune cell; CPS: combined positive score; ND: not described; TIL: tumor infiltrating lymphocyte; TAM: tumor associated macrophage; ES-SCLC: extensive-stage small cell lung cancer; PFS: progression-free survival; OS: overall survival; FOXP3: forkhead/winged-helix transcription factor 3.						

PD-L1在肿瘤细胞的表达是否有预后价值尚不明确，多数研究倾向其与更好的预后相关（表 1）^[20, 23, 38, 39]^。Yu等^[23]^对194例患者的分析显示肿瘤细胞PD-L1表达与更多的TIIC浸润相关，广泛期患者表达阳性者OS有延长趋势，但PD-L1表达与分期无相关性。Bonanno等^[20]^检测104例SCLC标本显示PD-L1表达在Ⅰ期-Ⅲ期高于Ⅳ期，单因素分析显示其与更长的OS相关，多因素分析无显著差异。Inamura等^[39]^对74例患者的分析显示PD-L1阳性者具有更低的肺癌特异性死亡率和总死亡率。Ishii等^[38]^对102例患者的分析显示PD-L1阳性患者更多为局限期且具有更长的OS。Carvajal- Hausdorf等^[19]^的研究显示肿瘤细胞表达PD-L1与5年生存率无关，其采用定量免疫荧光检测，而非其他研究采用的免疫组化，且检测出的肿瘤细胞PD-L1阳性率非常低（7.3%）。只有一项研究^[40]^显示PD-L1表达与更差的生存相关。研究结论差异的原因不清，可能与使用的PD-L1抗体和定义表达阳性的阈值不同、标本取材存在差异等因素有关。

关于肿瘤细胞表达PD-L1的ICI疗效预测价值的研究缺乏，因此结论尚不明确。Ⅰ期/Ⅱ期CheckMate 032研究显示PD-L1阳性和阴性患者使用纳武利尤单抗的ORR无显著差异^[10]^。在Ⅲ期CASPIAN研究中，PD-L1在肿瘤细胞的表达不影响度伐利尤单抗联合化疗的疗效和生存时间^[41]^。

#### 2.4.2 PD-L1在SCLC肿瘤浸润免疫细胞的表达

虽然总体而言PD-L1在肿瘤细胞的表达与PD-(L)1抑制剂疗效相关，但一些PD-L1阳性者无效，其中一个可能原因是PD-L1在肿瘤细胞的表达除了受到T细胞产生的干扰素γ诱导，也可能会受到肿瘤细胞内在机制如原癌基因信号的影响，而只有前者才是肿瘤适应性免疫抵抗的表现，因此更具有预测疗效价值^[34, 42]^。对多种类型肿瘤的检测显示PD-L1不仅表达于肿瘤细胞，还表达于以巨噬细胞为主的各种TIIC ^[23, 42]^，可能更能代表肿瘤产生了适应性免疫抵抗，对负瘤小鼠和人类多瘤肿标本的检测显示PD-L1表达于TIIC比表达于肿瘤细胞更能预测PD-(L)1抑制剂的疗效^[42]^。

和其他瘤种相符的是，多数研究^[20, 23, 24, 26, 41]^显示PD-L1在SCLC的TIIC（包括单核-巨噬细胞、淋巴细胞和树突状细胞等）比肿瘤细胞表达阳性率更高，为18.5%-56.3%（表 1）：Schultheis等^[26]^对94例SCLC或肺外小细胞癌的检测发现肿瘤细胞不表达PD-L1，但肿瘤与间质交界处的巨噬细胞PD-L1阳性率为18.5%。Ⅲ期CASPIAN研究显示277例标本中PD-L1在肿瘤细胞的阳性率为5.1%，在免疫细胞的阳性率为22.3%^[41]^。

关于TIIC PD-L1阳性与SCLC患者生存关系的有限研究提示其可能与更好的预后相关（表 1），这与肿瘤细胞PD-L1阳性的预后价值相似：Berghoff等^[43]^对32例脑转移SCLC患者的研究显示TIL PD-L1表达与更长的生存相关；Bonanno等^[20]^对104例SCLC标本检测显示PD-L1在肿瘤细胞和TIIC的表达与更早分期有显著相关性，单因素分析显示其与更好的生存相关，多因素分析无显著差异。

现有的研究提示PD-L1在TIIC表达可能具有预测PD-1抑制剂疗效的价值，但因为缺乏大样本研究，结论尚不肯定：Ⅱ期KN158研究显示PD-L1阳性患者使用帕博利珠单抗的疗效优于PD-L1阴性患者，阳性和阴性患者的ORR分别为35.7%（15/42）和6%（3/50），OS分别为14.6个月和7.7个月^[44]^。而另一项Ⅰ期/Ⅱ期CM032研究^[10]^中纳武利尤单抗的ORR与PD-L1表达无关，阳性和阴性患者分别为9%（1/11）和14%（9/64）。这两项研究结果的不同不排除与PD-L1检测细胞群体不同相关：前者检测的是肿瘤细胞、淋巴细胞和巨噬细胞，称为综合阳性评分（combined positive score, CPS），后者检测的仅为肿瘤细胞^[10, 44]^。Gadgeel等^[24]^的研究显示8例/20例SCLC TIIC PD-L1阳性，阳性细胞主要是围绕肿瘤巢周围分布的巨噬细胞，TIIC的PD-L1表达与帕博利珠单抗维持治疗后更长的PFS和OS相关。当PD-L1抑制剂与化疗联合治疗SCLC时，PD-L1在TIIC的表达与ORR和OS无相关性^[41]^。

总之，目前多数研究显示PD-L1在SCLC肿瘤组织表达水平偏低，与其他瘤种相似的是其阳性率在TIIC高于肿瘤细胞。PD-L1在肿瘤细胞和TIIC的表达具有相关性^[23, 40]^（表 1）。多数研究显示PD-L1在肿瘤细胞和TIIC的表达倾向与更好的预后相关。关于PD-L1表达对ICI疗效预测价值的有限研究提示PD-L1在肿瘤细胞的表达可能与疗效无明显相关性，而其在TIIC的表达可能有一定的疗效预测价值，但由于缺乏大规模研究的数据，结论尚不清楚。而当PD-L1抑制剂与化疗联合时，PD-L1在肿瘤细胞和免疫细胞的表达均与疗效无明显相关性。

### 2.5 SCLC的MHC Ⅰ和MHC Ⅱ下调

#### 2.5.1 MHC Ⅰ和MHC Ⅱ在肿瘤细胞表达的意义

肿瘤细胞表面的MHC Ⅰ分子能够将肿瘤抗原肽提呈给CD8^+^ T细胞，引发CTL反应^[45]^。多种类型肿瘤的MHC Ⅰ蛋白表达缺失或下降，造成其不能向CD8^+^ T细胞提呈抗原肽^[45]^。另外，MHC Ⅰ在肿瘤细胞下调还可能是造成T淋巴细胞不能浸润肿瘤内部的原因之一^[46]^。MHC Ⅱ能够将抗原肽交叉提呈给CD4^+^辅助T细胞，参与抗肿瘤免疫反应如激活抗原特异性免疫效应细胞、维持CTL活性、招募巨噬细胞等固有免疫细胞浸润、释放各种炎性因子和参与免疫记忆形成等^[47]^。细胞因子能够诱导癌细胞表达MHC Ⅱ^[45]^，继而使之具有激活辅助T细胞的作用^[45]^。

#### 2.5.2 MHC Ⅰ和MHC Ⅱ在SCLC表达下调

与NSCLC相比，SCLC的细胞系和肿瘤组织标本的MHC Ⅰ和/或β2-微球蛋白表达减少^[45]^。He等^[48]^检测了42个SCLC细胞系、55个NSCLC细胞系和278例肺癌标本中癌细胞和TIL的MHC Ⅱ表达。结果显示SCLC细胞系和肺癌标本肿瘤细胞无MHC Ⅱ表达，而12.7%的NSCLC细胞系MHC Ⅱ表达阳性。SCLC组织TIL的MHC Ⅱ表达水平也显著低于NSCLC。研究者提出MHC Ⅱ在SCLC下调可能是发生免疫逃逸的原因之一。Yazawa等^[49]^得到同样结果：未检测到人类白细胞抗原DR（human leukocyte antigen-DR, HLA-DR）蛋白在任何SCLC细胞表达，而在NSCLC细胞有不同程度表达。

*MHC Ⅰ*基因在SCLC细胞没有缺失或重组，提示表达在mRNA转录环节发生了阻滞。Ⅱ类反式激活因子（class Ⅱ transactivator, CIITA）是介导IFN-γ诱导MHC Ⅰ和MHC Ⅱ表达的重要转录因子之一^[45, 49]^。研究^[49]^显示MHC在SCLC表达下调是由于严重的CIITA表达降低引起。进一步的机制研究显示具有碱性螺旋-环-螺旋（basic helix-loop-helix, bHLH）结构的转录因子包括HASH-1和L-Myc在SCLC过表达^[45]^，其能够竞争性结合于*CIITA*基因启动子区的E-box上，抑制IFNγ诱导的CIITA表达，继而造成MHC表达受抑^[45, 49]^。

### 2.6 SCLC患者外周血免疫细胞改变

CD8^+^ T细胞是参与抗肿瘤免疫的重要免疫细胞亚型。An等^[9]^对未治疗ES-SCLC患者外周血T细胞亚群的检测显示CD8^+^ T细胞增殖受抑制，多因素分析显示CD8^+^ T细胞抑制程度与患者接受标准治疗后更短的PFS显著相关，提示SCLC患者外周血淋巴细胞免疫能力受损，并且可能影响生存。另一方面，几项研究^[30, 31]^均显示具有抑制抗肿瘤免疫作用的MDSC在SCLC患者外周血数量和比例增加并且与更差的预后相关。

## 3 总结

ICI虽然在SCLC药物治疗领域取得了突破性进展，但相比较其他对免疫治疗敏感的瘤种疗效仍不令人满意。如本综述所示，已有一些研究提示SCLC存在多样的免疫抑制机制，如肿瘤组织中存在以巨噬细胞为主的免疫抑制细胞浸润，肿瘤低水平表达PD-L1和MHC分子，患者外周血免疫细胞发生有利于肿瘤生长的改变，这些因素可能限制了ICI在SCLC的疗效，但相关研究仍然缺乏，应进行更多研究探讨这些问题，这有利于采取针对性治疗以提高ICI的疗效。另外，如本综述所示，PD-L1阳性免疫细胞特别是巨噬细胞在肿瘤的浸润以及免疫细胞在肿瘤内的分布位置可能更能反映适应性免疫抵抗的存在，应进一步开展回顾性和前瞻性研究探讨这些指标与ICI疗效的关系，这可能有利于更准确筛选出对ICI敏感的SCLC患者。
